# Hospital-Acquired Condition Rate of Admitting Facility Does Not Predict Mortality in Traumatically Injured Patients

**DOI:** 10.7759/cureus.23908

**Published:** 2022-04-07

**Authors:** Christopher B Horn, Joseph F O'Malley, Evan P Carey, John T Culhane

**Affiliations:** 1 Trauma/Critical Care, University of Cincinnati Medical Center, Cincinnati, USA; 2 General Surgery, Saint Louis University School of Medicine, St. Louis, USA; 3 Data Scientist, Denver–Seattle Center of Innovation for Veteran-Centered and Value-Driven Care, Veterans Affairs Eastern Colorado Health Care System, Aurora, Colorado, Denver, USA; 4 Surgery, Saint Louis University School of Medicine, Saint Louis, USA

**Keywords:** hospital-acquired conditions (hacs), catheters, hospital aquired infection, cauti prevention, cauti, central line-associated infections (clabsi), hospital quality, trauma, hap, hospital acquired conditions

## Abstract

Background: Hospital-acquired conditions (HACs) are increasingly scrutinized as markers of hospital quality and are subject to increasing regulatory and financial pressure. Despite this, there is little evidence that HACs are associated with poor outcomes in traumatically injured patients, or that lower HAC rates are a marker of a better quality of care. Our study compares mortality rates in hospitals with high versus low rates of HAC. Our hypothesis is that high HAC trauma centers have higher mortality.

Methods: The latest editions of the National Trauma Data Bank (NTDB) containing facility identification keys (2011 to 2015) were combined. The HACs targeted by the Centers for Medicare and Medicaid Services (CMS) Hospital-Acquired Condition Reduction Program (HACRP) were identified. Hospital-acquired conditions per 1000 patient-days were calculated for individual trauma centers, and these facilities were stratified into quartiles by HAC rate. Propensity score matching was used to match patients admitted to hospitals in the highest versus the lowest quartiles.

Results: Complete data was available for 3,510,818 patients; 58,296 (1.67%) developed HACs recorded in the NTDB. Good performing centers had a mean of 0.84 HACs per 1000 patient-days compared to 7.82 at poor-performing centers. After propensity matching, patients treated at good performing centers had higher mortality of 1.22% versus 1.02% at poor-performing centers (p<0.001). The facility characteristics most over-represented in the poor performing quartile were: University (45.19% vs 10.59%, p<0.001), American College of Surgeons (ACS) Level I Status (31.85% vs 2.24%, p<0.001), and bed size > 600 (28.15% vs 5.5%, p<0.001).

Conclusion: Injured patients treated at poor-performing centers (high HAC) have reduced mortality relative to good performing centers (low HAC). Large academic centers were overwhelmingly represented in the poor-performing quartile. Hospital-acquired conditions may be markers of a non-modifiable underlying patient and facility characteristics rather than markers of poor hospital quality.

## Introduction

In 2000, the National Institutes of Health (NIH) released “To Err is Human: Building a Safer Healthcare System”. In this report, the authors argued that between 44,000 and 98,000 patients a year die due to potentially preventable errors [1]. Since its release, the NIH has identified a list of hospital-acquired conditions (HAC) which they believe can reasonably be prevented with proper risk reduction measures. These include infectious complications such as central-line associated bloodstream infections (CLABSI), catheter-associated urinary tract infections (CAUTI), surgical site infections (SSI), and ventilator-associated pneumonia (VAP). Non-infectious HACs targeted for reduction include pressure ulcers, deep venous thrombosis (DVT), and pulmonary embolism (PE) [2]. These HACs are increasingly scrutinized by regulatory and reimbursement agencies.

In response to congressional mandate through the Medicare Modernization Act of 2003 and the Deficit Reduction Act of 2005, on October 1, 2008, the Centers for Medicare & Medicaid Services (CMS) initiated non-payment for certain conditions which were not present on admission [3]. In addition, the Center for Medicare and Medicaid Services (CMS) began factoring HACs into their calculations for publicly available hospital quality metrics [4]. In August 2013 the Hospital-Acquired Condition Reduction Program (HACRP) under the Affordable Care Act, was announced. Beginning in October 2014, the hospitals in the highest quartile of HAC, have been penalized 1% of their total Medicare payment [5].

Hospital-acquired conditions have diminished following these changes, but there is little evidence this has resulted in an improvement in clinically important patient outcomes [6-10]. The HAC reduction may not always result in better outcomes, but the HAC rate could still be a marker of an institution’s overall quality of care. To better elucidate the relationship between HAC rates and hospital quality, we performed an analysis of the National Trauma Data Bank (NTDB) to examine the relationship between HAC rates and mortality. We hypothesized centers with lower rates of HAC (good performing) would have lower mortality than hospitals with higher rates of HAC (poor performing).

## Materials and methods

This is a retrospective cohort study examining the association of the HAC rate of trauma centers with mortality. The 2011 to 2015 editions of the NTDB were used because these are the latest years that include a facility identification key. The Elixhauser index with van-Walraven modification was used to summarize baseline comorbidity [11,12]. Revised trauma scores were calculated as a physiologic index of trauma severity [13]. The injury severity score (ISS) was used as an anatomic injury scale. Payment type was included as an indirect measure of socioeconomic status. Baseline characteristics were reported for the entire population.

Patients with a hospital length of stay (LOS) of less than two days were excluded as this is generally insufficient time for a patient to develop a HAC. The NTDB does not report CAUTI for the years analyzed. We chose to examine UTIs with indwelling urinary catheter placement as a surrogate for CAUTI. This variable, which we call catheter exposed UTI, is defined as an International Classification of Diseases, Ninth Revision (ICD-9) code of 599.0 (urinary tract infection) in a patient with an ICD-9 procedure code of 57.94 (indwelling urinary catheter placement). Individual HACs were combined into a composite measure of HAC for each facility. The HACs included in the composite measure are CLABSI, SSI, pressure ulcer, DVT, and PE. Since catheter exposed UTI is not identical to CAUTI, it was analyzed separately but not included in the composite measure.

The HAC rates were calculated as HAC episodes per 1000 patient-days. Hospitals were divided into quartiles based on their HAC rates. To adjust for baseline differences between high and low HAC rate hospitals, a propensity match was implemented between the highest and lowest quartiles. Multivariate logistic regression was used to calculate a propensity score with the outcome of treatment at a hospital in the top-performing quartile. Demographic variables included were age, gender, race, and ethnicity. Trauma-related variables include mechanism of injury, ISS, and revised trauma score. Comorbidities include alcohol abuse, bleeding disorder, stroke, chronic respiratory disease, liver disease including cirrhosis, cancer, dementia, diabetes, drug abuse, myocardial infarction, peripheral vascular disease, hypertension, psychiatric disorder, chronic glucocorticoid use, obesity, and smoking. Payment status variables include Medicaid, Medicare, private insurance, and self-pay.

Missing data were handled with list-wise deletion. A calliper width of 0.2σ was used. A greedy nearest neighbor algorithm was then used to generate matches between the top and bottom quartile hospitals in a one-to-one ratio without replacement. Standardized difference of means (SDM) was calculated for baseline variables before and after the match. The SDM is the arithmetic difference between the means divided by the population standard deviation. Binary categorical variables were coded as one or zero, and means were calculated using these values.

Characteristics of facilities in propensity-matched cohorts from the best and worst-performing quartiles were calculated and compared. Mortality was compared between quartiles for each HAC separately and the composite. We chose mortality as the dependent variable because it is the most important outcome and as a simple and unmistakable binary condition, it is the least affected by reporting bias. Chi-square and Student’s T-Test were used to test for the significance of categorical and continuous variables respectively.

This study was deemed exempt by the institutional review board from approval because we used publicly available de-identified data. All statistical analysis was conducted with Statistical Package for the Social Sciences (SPSS), version 26.0 (IBM Corp., Armonk, N.Y., USA). Results are reported as per the Strengthening the Reporting of Observational Studies in Epidemiology (STROBE) guidelines 13.

## Results

Complete data were available for 3,510,818 patients. Most patients were young (mean 45.2) and male (63.4%) with few comorbidities; the average Elixhauser index was 0.31. Full baseline characteristics are shown in Table 1. 

**Table 1:** Baseline Characteristics UTI: Urinary tract infection, CLABSI: Central-line-associated bloodstream infections, SSI: Surgical site infections, DVT: Deep venous thrombosis, PE: Pulmonary embolism, HAC: Hospital-acquired condition, AKI: Acute kidney injury, SD: Standard deviation, n: number

Characteristic	n or Mean	% or Standard Deviation
Male Sex (n/%)	2225703	63.42%
Age (Mean/SD)	45.23	24.17
Penetrating (n/%)	314820	8.97%
ISS (Mean/SD)	9.06	8.34
Revised Trauma Score (Mean/SD)	7.55	1.07
Medicaid (n/%)	478954	13.65%
Medicare (n/%)	784366	22.35%
Private (n/%)	836652	23.84%
Self-Pay (n/%)	511473	14.57%
Elixhauser Index (n/%)	0.31	2.81
Alcoholism (n/%)	256377	7.3%
Bleeding Disorder (n/%)	172236	4.91%
Cancer (n/%)	25143	0.72%
Congestive Heart Failure (n/%)	94691	2.7%
Smoking (n/%)	546875	15.58%
Chronic Renal Failure (n/%)	29529	0.84%
Stroke (n/%)	66868	1.91%
Diabetes Mellitus (n/%)	359736	10.25%
Angina (n/%)	5545	0.16%
Myocardial Infarction (n/%)	38596	1.1%
Peripheral Vascular Disease (n/%)	13825	0.39%
Hypertension (n/%)	885558	25.23%
Chronic Respiratory Disease (n/%)	246982	7.04%
Steroid Use (n/%)	15204	0.43%
Cirrhosis (n/%)	23233	0.66%
Dementia (n/%)	72344	2.06%
Psychiatric Disorder (n/%)	199952	5.70%
Drug Abuse (n/%)	139254	3.97%
Obesity (n/%)	143282	4.08%
Documented Foley Placement (n/%)	37672	1.07%
UTI (n/%)	40702	1.16%
Foley Exposed UTI (n/%)	1296	0.04%
CLABSI (n/%)	2923	0.08%
Decubitus Ulcer (n/%)	14224	0.41%
SSI (n/%)	13817	0.39%
DVT (n/%)	24341	0.69%
PE (n/%)	10254	0.29%
Any HAC (n/%)	58296	1.67%
Mortality (n/%)	42058	1.20%
AKI (n/%)	16206	0.46%
Return to ICU (n/%)	18201	0.52%
Length of Stay (Days) (Mean/SD)	4.5	7.56
ICU Days (Mean/SD)	1.34	4.35
Ventilator Days (Mean/SD)	0.62	3.39
Adjusted UTI Rate (n/%)	2.56	2%
Foley Exposed UTI Rate (n/%)	0.08	0.24%
Adjusted CLABSI Rate (n/%)	0.18	0.33%
Adjusted Decubitus Ulcer Rate (n/%)	0.9	0.85%
Adjusted Surgical Soft Tissue Infection Rate (n/%)	0.85	0.73%
Adjusted Deep Venous Thrombosis Rate (n/%)	1.58	1.53%
Adjusted Pulmonary Embolism Rate (n/%)	0.66	0.46%

The aggregated NTDB contained patients from 963 centers. The good performing quartile contained 254 centers and there were 173 centers in the poor performing quartile. Characteristics of good and poor performing centers are shown in Table 2.

**Table 2:** Characteristics of good performing and poor performing centers based on adjusted composite HAC rate Chi-square and Student’s T-Test were used to test for the significance of categorical and continuous variables respectively. ACS: American College of Surgeons, HAC: Hospital-acquired condition, SD: Standard deviation, n: number

	Good Performing Facility		Poor Performing Facility		
	Mean	SD	Mean	SD	P
Adjusted Composite HAC Rate	0.84	0.82	7.82	3.99	<0.001
ACS Level	n	%	n	%	
I	11	2,24	43	31.85	<0.001
II	44	8.96	30	22.22	<0.001
III	55	11.2	3	2.22	0.001
Missing	211	42.97	20	14.81	
N/A	170	34.62	39	28.89	
Teaching Status	n	%	n	%	
Community	138	28.11	43	31.85	0.395
Non-Teaching	131	26.68	15	11.11	<0.001
University	52	10.59	61	45.19	<0.001
Missing	170	34.62	16	11.85	
Bed Size	n	%	n	%	
Beds <= 200	98	19.96	17	12.59	0.050
Beds 201-400	147	29.94	33	24.44	0.212
Beds 401-600	49	9.98	31	22.96	<0.001
Beds >600	27	5.5	38	28.15	<0.001
Missing	170	34.62	16	11.85	

Prior to propensity matching, 864,251 patients treated at poor-performing centers were compared with 878,748 at good performing centers. After propensity matching for the composite HAC rate, 791,093 patients were included for both good and poor performing centers, representing a successful match of 90.8% of patients. All post-match standardized mean differences were less than 10%. Pre and post-match baseline characteristics are included in supplementary tables 4, 5 (see Appendix A).

The mortality of patients for each subgroup treated at good and poor performing centers concerning the listed HACs is shown in Table 3. Mortality in the facility with the highest rate of each HAC (e.g., UTI) is compared with mortality in the facility with the lowest rate. The composite HAC is the sum of all reported HACs. Mortality is for all patients admitted to the facility in the specified HAC quartile, not only the patients with the HAC.

**Table 3:** Mortality in good versus poor-performing centers stratified into worst and best quartiles by each HAC, followed by the composite of all reported HACs. Chi-square and Student’s T-Test were used to test for the significance of categorical and continuous variables respectively. Cath: Catheter, UTI: Urinary tract infection, CLABSI: Central-line associated bloodstream infections, HAC: Hospital-acquired condition, SSI: Surgical site injury, DVT: Deep venous thrombosis, PE: Pulmonary embolism

	Admitted to Poor Performing Facility for the Listed HAC		Admitted to Good Performing Facility for the Listed HAC		
HAC	n	%	n	%	P
UTI	7186	1.04	7986	1.16	<0.001
Cath Exposed UTI	9712	1.23	11045	1.4	<0.001
CLABSI	8807	1.2	8549	1.17	0.049
SSI	7692	1.08	8361	1.17	<0.001
Pressure Ulcer	8071	1.09	8936	1.2	<0.001
DVT	7382	1.0	8714	1.18	<0.001
PE	7083	0.99	8669	1.22	<0.001
Composite HAC	7194	1.02	8601	1.22	<0.001

The higher number of cases of catheter exposed UTI is a consequence of the much lower number of patients with this condition. This resulted in a greater calliper width in the propensity match with fewer unmatched patients. Mortality for catheter exposed UTI patients was also clustered into the highest and lowest quartiles, with fewer in the middle quartiles which were dropped.

## Discussion

The Agency for Healthcare Research and Quality (AHRQ) National scorecard shows the incidence of HACs targeted by payment for performance is decreasing in US hospitals. Between 2014 and 2017 the targeted HAC rate decreased from 99 to 86 per 1000 discharges [6]. Despite this reduction, there is evidence that payment penalties have not improved clinical outcomes. Arntson et al. showed the rate of 30-day mortality did not decline after the implementation of the HACRP [7]. Sankaran et al. compared hospitals penalized under HACRP versus those not penalized. The mortality for each was identical at 9%. Penalties did not result in subsequent improvement of complication rates or clinically important outcomes [8]. Ryan et al. examined the correlation of hospital quality with performance-based payment adjustments under Medicare’s Hospital Value-Based Purchasing Program (HVBP). They found that HVBP was not associated with improvement in patient experience or mortality [9]. Sheetz et al. found that measures targeted for improvement by the HARCP did not improve more than non-targeted measures, thus the HACRP program was not independently associated with improved patient outcomes [10].

Other studies offer possible explanations for the lack of correlation between targeted HACs and outcomes. There have been concerns that financial incentives to reduce HAC may lead to inappropriate clinical decisions. A survey study of 317 hospitals reported that the majority have instituted clinical changes in response to CMS policy. These include early removal of urinary and central venous catheters, routine cultures on admission, and shifting of resources from non-targeted to targeted HACs [14]. In general, measures taken to reduce HACs are likely positive, however, financial pressure may lead to unintended consequences. For instance, clinicians are encouraged to remove indwelling urinary catheters early to decrease CAUTI. In some cases, premature removal of catheters may lead to urinary retention and associated complications [15].

Detection of complications is subject to surveillance bias. Surveillance bias occurs when the outcome depends on the degree of screening and detection rather than the underlying prevalence of illness. Institutions have different thresholds for screening and diagnostic testing, and higher quality institutions may conduct a more extensive and accurate investigation. A systematic review of surveillance bias in outcome measures concluded that this issue particularly affects venous thromboembolism (VTE) diagnosis. They found that six trauma-related and two post-operative VTE studies reported evidence of surveillance bias [16]. Previous work on VTE reported a paradoxical relationship between VTE and outcome measures similar to what we found in our analysis - high-quality centers had higher rates of VTE than low-quality centers [17]. They hypothesized that hospitals with higher quality were better at detecting complications, resulting in higher reported VTE rates, despite better compliance with VTE prophylaxis.

Coding practices may have changed in response to the HACRP. The coding of complications is inherently subjective and to avoid financial penalties hospitals may under-report complication rates. Winters et al.conducted a review of studies that measured the positive predictive value of the HACRP administrative database using direct chart review as the reference standard. They found an extensive discrepancy between the two, generally resulting from coding error [18]. Fuller et al. found a clustering of HACRP scores around the penalty threshold and correlation with hospital characteristics such as teaching status. These patterns disappeared with an alternative classification system [19]. Sheetz et al. reported that HAC rates reported in a statewide clinical registry were much higher than those in the database used to determine HACRP rates and their attendant penalties [20].

The HACRP may be biased against large academic and safety-net institutions. Sankaran et al. showed hospitals penalized under the HACRP were more likely to be large academic medical centers with a higher proportion of disadvantaged patients [8]. Rajaram et al. found that hospitals penalized under HACRP were more likely to be Joint Commission accredited, major teaching hospitals, safety-net hospitals, and hospitals that cared for more complex patients. These hospitals were penalized despite having superior publicly reported outcome measures [21]. We found that this pattern was very pronounced in the trauma population. For instance, when comparing the composite of HACs, ACS Level I facilities are 14.2 times more likely to be in the poorest performing quartile. This may be related to the regionalization of the trauma system. Sicker and more socio-economically disadvantaged trauma patients tend to be transferred to larger academic centers [22,23]. A study of the NIS database showed the strongest risks for HAC were non-modifiable factors such as functional status and chronic disease [24]. Our model was adjusted for demographic and clinical baseline imbalance, but the available indices of comorbidity may not capture all the increased risk, making it impossible to eliminate all potential bias.

Another explanation was recently offered by Ladhani et al. They performed a secondary analysis of the 2016 Trauma Quality Improvement Project to determine the effects of CAUTI on traumatically injured patients. In propensity-matched analysis, patients who developed CAUTI had longer hospital LOS, ventilator days, ICU days, and more complications, but overall lower mortality rates. They hypothesized that rather than representing poor quality care, CAUTI rates potentially represented the unintended consequence of aggressive rescue interventions in these patients. Overall, the increased interventions resulted in increased LOS but decreased mortality. As such, the placement of a urinary catheter represented more effective care, but at the cost of an increased likelihood of CAUTI. Ladhani et al. hypothesized that due to urinary catheterization being a necessary component of rescue care, CAUTIs may represent a “necessary evil” [25].

Implications for policy

The HACRP legislation (Affordable Care Act section 3008) imposes a penalty of 1% Medicare inpatient payment on the worst-performing quartile based on the HAC rate [5]. However, our study shows trauma mortality is lower in the worst adjusted HAC quartile compared to the best, thus it is difficult to argue these facilities provide an inferior standard of care. This raises the question of whether it is appropriate to levy penalties against hospitals with better outcomes. An alternative is to measure compliance with best preventative practices but recognize that outcomes depend on many variables, some of which cannot be controlled. The concept of “never events” is not realistic. It may encourage inaccurate reporting, adversely influence clinical decisions, and punish academic and safety-net hospitals.

Strengths

Our study is the first to compare outcomes in high versus low performing HAC quartiles in trauma. By stratifying patients by admission to a good or poor performing facility as the independent variable, we can examine not only whether HACs correlate with patient outcomes, but also whether HACs are a marker of overall quality of care. 

Limitations

Our study is subject to errors inherent in large datasets, including incomplete and inaccurate data. As the NTDB is a convenience sample of trauma patients primarily at Level I and II trauma centers, the current results may be less applicable to non-trauma centers. We were unable to use the National Healthcare Safety Network definition of CAUTI due to the inability to determine the temporal relationship between catheter insertion and diagnosis of UTI. We believe that our surrogate variable of catheter exposed UTI is similar to CAUTI, but we recognize it is not identical, leading to our decision to omit it from our composite score of HACs. After 2015 the NTDB no longer includes facility identification keys, which prevented us from using more recent data.

## Conclusions

Patients admitted to trauma centers in the worst-performing quartile for adjusted HAC rate had lower mortality than patients admitted to centers in the best performing quartile. This suggests that HACs are not an accurate measure of the quality of care in trauma patients. The HAC rate is more likely a marker of comorbidity and socioeconomic status. Better patient risk stratification and alternative measures of quality should be developed to avoid penalizing the hospitals that care for the sickest patients.

## Appendices

Appendix A

**Table 4:** Prematch baseline comparison between poor and good performing composite HAC quartiles. Standardized difference of means calculated as described in the methods. HAC: Hospital-acquired condition, SD: Standard deviation, D/O: Disorder, CHF: Congestive heart failure, CRF: Chronic renal failure, MI: Myocardial infarction, PVD: Peripheral vascular disease, COPD: Chronic obstructive pulmonary disease, ISS: Injury severity score, RTS: Revised trauma score

	Poor Performing		Good Performing		
	n	%	n	%	%Std Diff
Male Sex	569251	65.87	532129	60.56	-11.02
White Race	577300	66.8	634914	72.25	11.86
Hispanic	86903	10.06	95826	10.9	2.78
Penetrating	92170	10.66	65823	7.49	-11.06
Medicaid	110458	12.78	112373	12.79	0.02
Medicare	169879	19.66	224109	25.5	13.99
Private	205353	23.76	214273	24.38	1.46
Self Pay	144781	16.75	117549	13.38	-9.43
Alcoholism	74475	8.62	47564	5.41	-12.57
Bleeding D/O	49584	5.74	31627	3.6	-10.13
Cancer	5004	0.58	5723	0.65	0.93
CHF	22112	2.56	24617	2.8	1.5
Smoking	159501	18.46	90682	10.32	-23.18
CRF	6960	0.81	7729	0.88	0.82
Stroke	17335	2.01	15742	1.79	-1.58
Diabetes	88886	10.28	87099	9.91	-1.24
Angina	2734	0.32	529	0.06	-5.96
MI	11907	1.38	7839	0.89	-4.58
PVD	3326	0.38	2853	0.32	-1.02
Hypertension	224621	25.99	205729	23.41	-5.98
COPD	61338	7.1	53758	6.12	-3.95
Steroid	4707	0.54	2354	0.27	-4.32
Cirrhosis	7460	0.86	4194	0.48	-4.76
Dementia	16506	1.91	17456	1.99	0.56
Psychiatric D/O	57364	6.64	31531	3.59	-13.86
Drug Abuse	46439	5.37	20666	2.35	-15.74
Obesity	46560	5.39	20422	2.32	-15.95
Characteristic		Mean		Mean	%Std Diff
Age		44.79		45.87	-4.45
ISS		9.86		8.05	21.84
RTS		7.49		7.62	-12.24
Elixhauser		0.18		0.41	-8.36

**Table 5:** Post-match baseline comparison between poor and good performing composite HAC quartiles. Standardized difference of means calculated as described in the methods. HAC: Hospital-acquired condition, SD: Standard deviation, D/O: Disorder, CHF: Congestive heart failure, CRF: Chronic renal failure, MI: Myocardial infarction, PVD: Peripheral vascular disease, COPD: Chronic obstructive pulmonary disease, ISS: Injury severity score, RTS: Revised trauma score

	Poor Performing		Good Performing		
	n	%	n	%	%SD
Male Sex	449290	63.83	456032	64.79	2.0
White Race	487404	69.25	477991	67.91	-2.88
Hispanic	75007	10.66	74008	10.51	-0.46
Penetrating	62114	8.82	62882	8.93	0.38
Medicaid	89879	12.77	88235	12.54	-0.7
Medicare	150914	21.44	131823	18.73	-6.76
Private	172712	24.54	184840	26.26	3.96
Self Pay	106909	15.19	105420	14.98	-0.59
Alcoholism	47317	6.72	45560	6.47	-1.01
Bleeding D/O	31591	4.49	31342	4.45	-0.17
Cancer	4217	0.6	4036	0.57	-0.34
CHF	17141	2.44	18223	2.59	0.99
Smoking	92400	13.13	89944	12.78	-1.04
CRF	5651	0.8	5265	0.75	-0.62
Stroke	12999	1.85	12502	1.78	-0.53
Diabetes	69726	9.91	65434	9.3	-2.07
Angina	2189	0.31	372	0.05	-6.0
MI	7518	1.07	7537	1.07	0.03
PVD	2422	0.34	2286	0.32	-0.33
Hypertension	172217	24.47	161445	22.94	-3.6
COPD	45517	6.47	44304	6.29	-0.71
Steroid	2559	0.36	2344	0.33	-0.52
Cirrhosis	4247	0.6	4038	0.57	-0.39
Dementia	13623	1.94	13549	1.92	-0.08
Psychiatric D/O	33032	4.69	30972	4.4	-1.41
Drug Abuse	22203	3.15	20608	2.93	-1.32
Obesity	21848	3.1	20413	2.9	-1.19
Characteristic		Mean		Mean	%SD
Age		44.78		43.36	5.92
ISS		8.83		8.62	2.67
RTS		7.57		7.58	-0.98
Elixhauser		0.34		0.36	-0.77
